# Risk factors and assessment for cardiovascular disease among HIV-positive patients attending a Nigerian tertiary hospital

**DOI:** 10.11604/pamj.2016.23.206.7041

**Published:** 2016-04-20

**Authors:** Ifeyinwa Dorothy Osegbe, Oyetunji Olukayode Soriyan, Abiola Ann Ogbenna, Henry Chima Okpara, Elaine Chinyere Azinge

**Affiliations:** 1 Department of Clinical Pathology, Lagos University Teaching Hospital, Idi-araba, Lagos state, Nigeria; 2 Department of Haematology and Blood Transfusion, Lagos University Teaching Hospital, Idi-araba, Lagos state, Nigeria; 3 Department of Chemical Pathology, University of Calabar Teaching Hospital, Cross-Rivers state, Nigeria

**Keywords:** Atherosclerosis, cardiovascular disease, HIV, risk assessment

## Abstract

**Introduction:**

Cardiovascular risk factors are prevalent in HIV-positive patients which places them at increased risk for cardiovascular disease (CVD). We aimed to determine the risk factors and risk assessment for CVD in HIV-positive patients with and without antiretroviral therapy.

**Methods:**

This was a cross-sectional study of HIV-positive patients attending the Lagos University Teaching Hospital, Nigeria. Anthropometric and blood pressure measurements were performed; fasting lipid profile, plasma glucose, homocysteine and hsCRP were determined, as well as prevalences and risk assessments. Statistical tests were used to compare the groups and p-value <0.05 was considered to be significant.

**Results:**

283 subjects were recruited for this study (100 HIV-positive treatment-naive, 100 HIV-positive treated and 83 HIV negative controls). Compared to the controls, mean (sd) values were significantly higher among HIV-treated subjects: waist circumference = 88.7 (10.4), p = 0.035; systolic bp= 124.9 (20.7), p = 0.014; glucose= 5.54 (1.7), p = 0.015; triglyceride= 2.0 (1.2), p < 0.001; homocysteine= 10.9 (8.9-16.2), p = 0.0003; while hsCRP= 2.9 (1.4-11.6), p = 0.002 and HDL-C = 0.9 (0.4), p = < 0.0001 were higher among the HIV-naïve subjects. Likewise, higher prevalences of the risk factors were noted among the HIV-treated subjects except low HDL-C (p < 0.001) and hsCRP (p = 0.03) which were higher in the HIV-naïve group. Risk assessment using ratios showed high risk for CVD especially in the HIV-naïve group. The median range for Framingham risk assessment was 1.0 - 7.5%.

**Conclusion:**

Risk factors and risk assessment for CVD are increased in HIV-positive patients with and without antiretroviral therapy. Routine evaluation and risk assessment for CVD irrespective of therapy status is necessary to prevent future cardiovascular events.

## Introduction

Cardiovascular disease (CVD) is a group of disorders of the heart and blood vessels which can manifest as coronary heart disease, cerebrovascular disease, peripheral arterial disease, rheumatic heart disease, congenital heart disease, deep vein thrombosis and pulmonary embolism [1]. It is a primary cause of death worldwide [2] with atherosclerosis being the most common pathological process that leads to it, involving a combination of vascular endothelial dysfunction, chronic inflammation, and dyslipidaemia [3]. According to the Global burden of disease study, current predictions estimate that by the year 2020, CVD, notably coronary heart disease (CHD), will become the leading global cause of total disease burden [4].

Several risk factors for CVD have been identified and can be categorized into two groups: Modifiable (by lifestyle and/or pharmacotherapy) and Unmodifiable. The traditional risk factors for CVD recognized by the current National Cholesterol Education Project (NCEP) Adult Treatment Panel III (ATP III) guidelines include [3]: age: men ≥45yrs, women ≥55yrs, cigarette smoking, hypertension or use of antihypertensive medication, dyslipidaemia, diabetes mellitus, obesity, family history of premature CHD in a first degree relative male ≤55yrs or female ≤65yrs, and prior CVD in the index individual.

In addition, a variety of novel biochemical markers have been suggested to identify individuals at increased risk for CVD, such as: markers of inflammation e.g. high sensitivity C-reactive protein (hsCRP)^5^, Homocysteine [6–8], markers of fibrinolytic and haemostatic function e.g. tissue type plasminogen activator antigen and fibrinogen respectively [9].

Sub-Saharan Africa is still scourged by the Human Immunodeficiency Virus (HIV) infection, and this remains a major health concern with a prevalence of 4.9% equating to 23.5 million people, of which 874,000 are plagued with infectious diseases such as tuberculosis [10]. But with the introduction of highly active antiretroviral therapy (HAART), HIV-infected patients are surviving AIDS-related causes of death [10]. Unfortunately, this population is faced with the new challenge of non-communicable diseases because the virus as well as HAART now predisposes them to endothelial dysfunction, chronic inflammation, and dyslipidaemia [11–13] which are necessary for atherosclerosis that may ultimately lead to CVD.

Metabolic abnormalities have been well-described in HIV positive patients on HAART especially those on Protease inhibitors. They include: hypertriglyceridemia, hypercholesterolemia, lipodystrophy and insulin resistance/type2 diabetes mellitus [14, 15], as well as fat redistribution, lipoatrophy or fat accumulation [16] referred to as HAART-associated morphologic and metabolic abnormality syndrome (HAMMAS) or HIV- associated lipodystrophy syndrome (HLS) [17]. These features can cause atherosclerotic CVD, therefore treated HIV patients may develop cardiovascular complications.

Similarly, untreated HIV positive patients are at risk for CVD. They have increased production of pro-inflammatory cytokines e.g. C-reactive protein, tumour necrosis factor, interleukin 6 (IL-6); with concomitant decrease in anti-inflammatory cytokines e.g. IL-10, adiponectin; which promotes endothelial activation and chronic inflammation [11, 12]. These are pathophysiological factors in the development of CVD. These cytokines can also have effects on different enzymes of lipid metabolism resulting in dyslipidaemia [13], a major CVD risk factor. Therefore, it is important to identify CVD risk factors and conduct risk assessments bearing in mind the differences in the aetiological processes of CVD among treated and untreated HIV positive patients. The outcome will be beneficial in prompting early intervention. The aim of this study was to determine the risk factors and assessment for CVD among HIV-positive patients attending a Nigerian tertiary hospital.

## Methods

### Study design

This was a cross-sectional study of cardiovascular disease risk factors in HIV-positive adult patients attending the HIV outpatient clinic of the Lagos University Teaching Hospital (LUTH), which is a tertiary hospital in Lagos, Southwest Nigeria. The study protocol was reviewed and approved by the Hospital's Research and Ethics Committee (ADM/ DCST/HREC/200).

### Study population

Male and female adult patients aged 21 to 60 years with confirmed HIV seropositivity by double enzyme linked immunosorbent assay (ELISA) and confirmatory Western blot were included as the subjects in the study. The sampling technique was by stratified, random sampling, where stratification was by whether patients had been taking antiretroviral therapy for over one year or not at all. Patients were randomly selected using a table of random numbers. Those with CD4+ T cell counts ≥350cells/mm^3^ and who had not received HAART were designated as the ‘naive’ group, while patients who had commenced HAART for at least six months were designated the ‘treated’ group.

Considering that there are several CVD risk factors with their respective prevalence rates [7, 18] the sample size was not calculated using the formula: n = z^2^pq/d^2^ where n = sample size, z = critical value at 95% confidence level, usually set at 1.96, p = Prevalence, q = 1-p, d = precision of 5% (0.05) [19]. Literature review showed that the sample size of 171 was used in a similar study [18].

But to increase the validity of our study, anextra 17% were included to cater for non-responders, giving a sample size of 200 for the subject cases. The controls were age and sex-matched adult HIV negative participants recruited during community outreaches after voluntary counseling and testing. They were included in acase: control ratio of 2:1; however some controls were lost to follow up. Patients with secondary causes of dyslipidaemia and hyperglycaemia were excluded, including pregnant women and nursing mothers. Participants were recruited for the study after they were fully informed and written consent obtained. Confidentiality was ensured.

### Data collection

Questionnaires were administered to the subjects to obtain basic demographic data and history of: HAART use- type and duration, cigarette smoking, antihypertensive and diabetic medication use. Thereafter, blood pressure was taken in the sitting position after five minutes of rest using a digital sphygmanometer. Weight in kilograms (kg) was measured to the nearest 0.1kg using a calibrated measuring scale, while height in metres (m) was measured to nearest 0.1m using a calibrated stadiometerfor the calculation of body mass index (BMI= weight/ (height)2, Waist circumference (WC) in centimetres (cm) was measured to the nearest 0.1cm using a tape rule at midway between the subcostal plane and the iliac crest. Hip circumference (HC) was also measured at the largest width and used to calculate the waist-to-hip ratio (WHR = WC/HC).

### Specimen collection

The participants were requested to return in the morning after an overnight 10-12hr fast when antecubital venous blood collection was performed. Five mls of blood was drawn into a plain vacutainer for lipid profile and hsCRP; 3mls into a fluoride oxalate vacutainer for glucose assay and 3mls into an EDTA vacutainer for homocysteine determination. The specimens were taken to the laboratory where they were centrifuged at 4000rpm for 10minutes. The supernatant, plasma or serum as the case may be, was separated out. The fluoride oxalate plasma was analyzed for glucose daily. While the EDTA plasma was aliquoted into a cryogenic storage tube for homocysteine, also the serum was aliquoted into two cryogenic storage tubes (one for lipid profile and hsCRP each) and stored at -70°C in a well-monitored freezer (NuAire, USA), for one month until the analyses were performed. Haemolysed, icteric and lipaemic samples were excluded.

### Biochemical analysis

Glucose oxidase method was used to estimate fasting plasma glucose concentration, Total cholesterol (TC), Triglyceride (TG) and High Density Lipoprotein-Cholesterol (HDL-C) were analyzed with standard enzymatic methods, while Low Density Lipoprotein-Cholesterol (LDL-C) was determined by a direct, homogenous assay on a Roche Hitachi 902 analyzer (Roche diagnostics, Germany). The LDL-C method was employed to circumvent the limitation of the routinely used Friedewald equation [20], which cannot be applied when TG >400mg/dl, as this may be seen in HIV patients on HAART. A sandwich, solid-phase ELISA was used to determine hsCRP concentrations (Diagnostic Automation Inc., USA), as well as homocysteine (Axis Shield, Germany) and read outs pectrophotometrically with a Biorad microwell reader (Biorad, USA).

### Quality control

Precision studies were carried out for the lipids and glucose using bovine precision sera (RANDOX, UK) level 2 (lot no 407SN, expiry date 2014/03) and level 3(lot no 324SE, expiry date 2013/04); and coefficient of variation (%CV) was calculated for within and between assay runs. Liquichek liquid control (BIORAD, UK) medium level was used to control the hsCRP assay(lot no 0906296, expiry date 2012-10), while a tri-level control set (Axis Shield, Germany) was used to quality control the homocysteine assay (lot no 802883611, expiry date 2012-08-08).

### Statistical analysis

Data from the completed questionnaires and laboratory results were categorized into: Subjects (HIV-positive naive, HIV-positive treated) and Controls (HIV negative). They were entered unto a spreadsheet (Microsoft Office Excel 2007) where BMI and CVD risk ratios were calculated. Ten-year risk assessment for CVD was performed using Framingham risk score calculator [21]. Statistical analyses were performed using SPSS version 20 (Chicago IL, USA). Kolmogorov-Smirnov test was used to test for normality and results for hsCRP and homocysteine were log-transformed. One-way ANOVA was used to compare the mean values between the three subgroups, while the Student's t-test was used for comparison of mean of continuous variables between the controls and each subject subgroup to distinguish their individual contributing differences. Prevalence and 95% confidence intervalsof high risk factors for CVD were performed. Z-test was performed to compare prevalences between the naïve and treated subjects. The level of statistical significance was established at p-value of <0.05.

## Results

Two hundred and eighty-three (283) participants consisting of 170 (60%) females and 113(40%) males were included in this study. The HIV naïve patients were 100 with mean ± sd age of 35.6 ± 8.1years, the HIV treated patients were 100 with mean ± sd age of 37.5 ± 7.8years, and the age- and sex-matched HIV negative controls were 83 with mean ± sd age of 36.6 ± 7.2 yrs. Of the treated patients, eighty-five (85) had been administered NRTI-based therapy, while 15 received PI-based regimen.

The mean values of CVD risk factor variables are shown in Table 1 highlighting significant differences between the 3 groups, while in Table 2 the means were compared between the controls and each subject subgroup. The prevalence of risk factor variables using cut off levels of high risk for CVD are shown in Table 3.

**Table 1:** Comparison of the mean values of CVD risk factors in subjects and controls

Modifiable ASCVD risk factor	parameter	HIV naïve (n = 100) Mean ± sd	HIV treated (n = 100) Mean ± sd	Controls (n =83) Mean ± sd	One way ANOVA p-value
Obesity	BMI (kg/m^2^)	25.6 ± 5.3	25.5 ± 4.5	25.3 ± 5.9	0.93
	Waist Circumference (cm)				
	Women	87.7 ± 9.9	88.7 ± 10.4	81.9 ± 17.6	0.001^+^
	Men	86.3 ± 13.9	88.9 ± 13.0	86.8 ± 5.9	0.26
	Waist-to-Hip Ratio				
	Women	0.83 ± 0.05	0.87 ± 0.07	0.82 ± 0.07	<0.001^+^
	Men	0.87 ± 0.07	0.88 ± 0.06	0.87 ± 0.05	0.42
Hypertension	SBP (mmHg)	121.5 ± 20.7	124.9 ± 20.7	114.8 ± 11.7	0.001^+^
	DBP (mmHg)	73.8 ± 12.8	75.4 ± 13.9	72.9 ± 10.7	0.40
Diabetes mellitus	Glucose (mmol/L)	4.9 ± 1.6	5.54 ± 1.7	4.7 ± 1.7	0.002^+^
Dyslipidaemia	TC (mmol/L)	4.7 ± 1.4	5.5 ± 1.4	5.1 ± 1.10	<0.001^+^
	TG (mmol/L)	1.4 ± 0.7	2.0 ± 1.2	1.1 ± 0.6	<0.001^+^
	LDL-C (mmol/L)	3.1 ± 1.2	3.3 ± 1.2	3.3 ± 1.1	0.39
	HDL-C (mmol/L)	0.9 ± 0.4	1.5 ± 0.6	1.4 ± 0.3	<0.001^+^
Inflammation	hsCRP (mg/L)^§^	2.9 (1.4 – 11.6)	1.9 (1.3 – 2.2)	2.2 (0.8 – 4.5)	<0.001^+^
Atherothrombosis	Homocysteine (umol/L)^§^	10.9 (8.5 – 13.6)	10.9 (8.9 – 16.2)	16.3 (13.3 – 20.7)	<0.001^+^

+ statistically significantat <0.05

§ median (interquartile range)

**Table 2:** Comparison of mean values of CVD risk factors of each subject subgroup versus controls

Modifiable ASCVD risk factor	Parameter	HIV naïve (n = 100) Mean ± sd	T-test p-value^γ^	HIV treated (n = 100) Mean ± sd	T-test p-value^γ^	Controls (n =83) Mean ± sd
Obesity	BMI (kg/m^2^)	25.6 ± 5.3	0.78	25.5 ± 4.5	0.88	25.3 ± 5.9
	Waist Circumference (cm)					
	Women	87.7 ± 9.9	0.06	88.7 ± 10.4	0.035^+^	81.9 ± 17.6
	Men	86.3 ± 13.9	0.89	88.9 ± 13.0	0.55	86.8 ± 5.9
	Waist-to-Hip Ratio					
	Women	0.83 ± 0.05	0.67	0.87 ± 0.07	0.005^+^	0.82 ± 0.07
	Men	0.87 ± 0.07	0.89	0.88 ± 0.06	0.83	0.87 ± 0.05
Hypertension	SBP (mmHg)	121.5 ± 20.7	0.11	124.9 ± 20.7	0.014^+^	114.8 ± 11.7
	DBP (mmHg)	73.8 ± 12.8	0.74	75.4 ± 13.9	0.38	72.9 ± 10.7
Diabetes mellitus	Glucose (mmol/L)	4.9 ± 1.6	0.50	5.54 ± 1.7	0.015^+^	4.7 ± 1.7
Dyslipidaemia	TC (mmol/L)	4.7 ± 1.4	0.30	5.5 ± 1.4	0.05	5.1 ± 1.10
	TG (mmol/L)	1.4 ± 0.7	0.019^+^	2.0 ± 1.2	<0.0001^+^	1.1 ± 0.6
	LDL-C (mmol/L)	3.1 ± 1.2	0.29	3.3 ± 1.2	0.20	3.3 ± 1.1
	HDL-C (mmol/L)	0.9 ± 0.4	<0.0001^+^	1.5 ± 0.6	0.48	1.4 ± 0.3
Inflammation	hsCRP (mg/L)^§^	2.9 (1.4 – 11.6)	0.002^+^	1.9 (1.3 – 2.2)	0.95	2.2 (0.8 – 4.5)
Atherothrombosis	Homocysteine (umol/L)^§^	10.9 (8.5 – 13.6)	0.001^+^	10.9 (8.9 – 16.2)	0.0003^+^	16.3 (13.3 – 20.7)

+ statistically significant at <0.05

§ median (interquartile range)

γ comparing mean value of each subgroup with controls using Student t-test

**Table 3:** Comparison of prevalence of high risk factors for CVD in HIV subjects

Modifiable ASCVD risk factor	Parameter	Cutoff level for CVD risk	HIV naïve (n = 100) Prevalence% (95% CI)	HIV treated (n = 100) Prevalence% (95% CI)	z-test p-value
Obesity	Body Mass Index	≥ 30kg/m^2^	13% (7 – 19%)	17% (10 – 25%)	0.43
	Waist Circumference				
	Women	> 88cm	18% (11 – 25%)	27% (19 – 34%)	0.13
	Men	> 102cm	6% (2 – 11%)	9% (4 – 15%)	0.42
	Waist-to-Hip Ratio				
	Women	> 0.85	26% (18 – 34%)	28% (20 – 35%)	0.75
	Men	> 0.9	13% (7 – 19%)	15% (9 – 20%)	0.68
Hypertension	Systolic BP	> 140 mmHg	12% (6 – 19%)	23% (15 – 31%)	0.04^+^
	Diastolic BP	> 90 mmHg	11% (6 – 19%)	20% (11 – 28%)	0.08
Diabetes mellitus	Glucose	> 7.0mmol/L	2% (1 – 4%)	9% (4 – 16%)	0.03^+^
Dyslipidaemia	Total Cholesterol	≥ 5.2mmol/L	34% (26 – 43%)	47% (38 – 55%)	0.06
	Triglyceride	≥ 1.7mmol/L	21% (14 – 29%)	24% (15 – 32%)	0.61
	LDL-C	≥ 3.3mmol/L	30% (22 – 39%)	37% (28 – 46%)	0.29
	HDL-C	< 1.0 mmol/L	42% (33 – 50%)	11% (5 – 17%)	<0.001^+^
Inflammation	hsCRP	≥ 2 mg/L	51% (42 – 59%)	36% (27 – 46%)	0.03^+^
Atherothrombosis	Homocysteine	> 12 umol/L	35% (26 – 44%)	39% (29 – 49%)	0.56

+ statistically significant at <0.05

Risk assessment for CVD using Grover's risk ratio LDL-C/HDL-C [22] revealed mean ± sd of HIV naïve 3.6 ± 2.5 and HIV treated 2.5 ± 1.4 which fall in the range of 3.3 – 3.7 that signifies increased risk of death from CVD [23]. Similarly, TC/HDL-C [22] showed mean ± sd of HIV naïve 5.9 ± 3.7 and HIV treated 4.8 ± 5.2 which are >5.6 that signifies high risk for CVD [24]. Atherogenic Index (log TG/HDL-C) [25] showed mean ± sd of HIV naïve +0.18 ± 0.3 and HIV treated -0.03 ± 0.4, where the more positive the ratio, the higher the risk for CVD, and vice versa [26].

Ten year risk assessment for CVD using Framingham risk score performed on patients that had ≥2 risk factors showed median (interquartile range) of HIV naïve women n = 6, 1.5% (1 - 3.75%); HIV naïve men n = 9, 6.5% (2 - 11.25%); HIV treated women n = 16, 1.0% (1.0 - 4.5%);and HIV treated men n = 18, 7.5% (4.25 - 16.5%). Three patient data sets were excluded because they exceeded the equation variables’ limits (age = 20 – 99years, TC= 130 - 320mg/dl, HDL-C= 20 – 100mg/dl, Systolic blood pressure= 90 – 200mmHg) [21].

## Discussion

In this study, the major independent risk factors for CVD were identified in the HIV positive patients, although their mean values were not exceptionally elevated as similarly reported by Oduola et al [27]. Despite this, measures of obesity (WC, WHR), diabetes mellitus (fasting plasma glucose), hypertension (systolic blood pressure), dyslipidaemia (hypertriglyceridaemia, low HDL-C), hsCRP, and homocysteine in the subjects were significantly higher than the HIV negative controls.

High TG with low HDL-C in HIV naïve subjects compared to the HIV negative controls was demonstrated by Rose et al [28]. This corroborates our findings and may suggest that HIV infection is associated with modified HDL metabolism re-directing cholesterol to the apo B-containing lipoprotein and likely reduces the functionality of reverse cholesterol transport [29]. Also, the presence of endothelial lipase and phospholipase A2 during the vascular inflammation induced by the viral infection results in low HDL-C [30], therefore a dyslipidaemic pattern is associated with HIV infection itself. On the other hand, the metabolic changes we observed in the HIV-treated patients were in line with the features of HAMMAS, with women having greater prevalence of obesity.

The prevalence of dyslipidaemia among HIV treated subjects observed in another Nigerian study were lower than ours except hypertriglyceridaemia. Theirs revealed hypercholesterolaemia =28%, elevated LDL-C= 24% and hypertriglyceridaemia =35%, using desirable cut-off values of TC-200mg/dl (5.2mmol/L), LDL-C-130mg/dl (3.3mmol/L), TG-150mg/dl (1.7mmol/L) respectively [31]. The difference to our finding of hypertriglyceridaemia = 24% may be explained by the specific HAART medication the patients may be taking.

Protease inhibitors (PIs) are antiretroviral drugs that target the catalytic region of HIV protease and prevent it from cleaving translated precursor polyprotein to individual proteins in order to form mature viral particles, resulting in non-infectious viral particles. However, this region is homologous with regions of two human proteins that regulate lipid metabolism: cytoplasmic retinoic-acid binding protein-1 (CRABP-1) and low density lipoprotein-receptor-related protein (LRP) [16], which would result in increased apoptosis of peripheral adipocytes, decreased pre-adipocyte differentiation, decreased triglyceride storage in adipose tissue, increased VLDL production, impaired hepatic chylomicron uptake and endothelial triglyceride clearance, resulting in hypertriglyceridaemia [16, 32].

Although patients on nucleoside reverse transcriptase inhibitors (NRTI) and non-nucleoside reverse transcriptase inhibitors (NNRTI) may develop lipodystrophy, insulin resistance, high TC and LDL-C, only modest elevations in TG have been described [33, 34]. The increased prevalence of hypertriglyceridaemia in HIV patients treated with PI versus the NRTI was also demonstrated in a study by Salami et al [35]. In our study, only 15 HIV treated patients were on PIs because it is second-line medication, administered when the NRTI-based regimen fails [36]. Higher mean hsCRP concentration and prevalence were observed in the HIV naïve subjects when compared to treated patients and the controls (p <0.001), similar to reports from other studies [37, 38]. This could be due to the unhindered chronic inflammation by the viral infection in these subjects which may be alleviated with HAART and antioxidants.

Plasma Homocysteine concentrations were significantly elevated in both naive and treated HIV subjects than the controls, corroborating findings in other studies [39, 40]. We demonstrated hyperhomocysteinaemia prevalence of HIV naïve= 35% and HIV treated = 39%; which were higher than what was observed in a study conducted in Italy, where the HIV treated prevalence was 28.3%, using the same cut-off value of > 12µmol/L [41]. This may be due to the reduced intake of folic acid, vitamin B6 and vitamin B12 supplements in our African population which are required for the metabolism of homocysteine.

### Risk assessment

It is recommended to examine for CVD traditional risk factors in adults 20 -79 years of age who are free of atherosclerotic CVD in a bid to assess their risk for future coronary heart disease [3]. Absolute cutoff values have been assigned for each risk factor (Table 3) which give an indication of high risk for CVD.

Although quantitative measurements of the lipid profile can be used to ascribe risk and target treatment strategies [3], Gotto *et al* claim that that approach is more helpful for extreme values and not for marginal values [42] like those seen in our subjects. The thirty-person/ten-country study recommends using apoB/apoA1 ratio to evaluate lipoprotein risk for CVD [43], but a more readily available correlate would be LDL-C/HDL-C which has proven to be the best lipid-related predictor of future cardiovascular event [22, 44] than LDL-C or HDL-C alone [45]. Unfortunately, these ratios have not been validated in HIV-infected populations and may be limited because it excludes TG which is a common feature in them, especially those on HAART. Therefore, atherogenic index (AI) which is derived from log10 of serum (TG/HDL-C) and has been shown to be a surrogate of small, dense LDL particle size that predicts coronary artery disease independently, as well as type 2 diabetes mellitus, high blood pressure and metabolic syndrome [25] may be useful.

Using these risk indices, we observed an increased risk for CVD, which was exaggerated in the HIV naïve group, corroborated by a study conducted in India [46]. Similarly, a study conducted in Uganda demonstrated increased TC/HDL-C ratio in the HIV naïve (4.6) versus the HIV treated (3.4) [47]. These findings are attributed to the relatively lower HDL-C levels, which have also been independently associated with increased CVD risk [3].

Although published guidelines do not recommend measurement of any of the emerging novel risk factors for the purpose of routine evaluation [48], nor for risk assessment [3]; hsCRP ≥ 2mg/L can be applied to revise risk assessment upward when risk-based treatment decisions are uncertain after quantitative risk assessments [48]. Similarly, in a meta-analysis of the association between homocysteine and CVD, it was found that for every 5umol/L increase in serum homocysteine concentration, the risk of ischaemic heart disease increased 20% to 30% [49]. Thus, clinically, the measurement of total homocysteine is considered important as a risk factor for CVD and other disorders [9, 50, 51].

The Framingham risk-assessment tool is a coronary prediction algorithm that provides estimates of total CHD risk (risk of developing one of the following: angina pectoris, myocardial infarction, or coronary disease death) over the course of 10 years [52]. It is used for individuals in the general population who have two or more risk factors for CVD. The factors used to estimate risk include: age, gender, TC, HDL-C, systolic blood pressure and antihypertensive medication, and cigarette smoking. Relative risk for CHD is estimated by comparison to low risk Framingham participants, and standard practice is to initiate some form of intervention when the 10-year Framingham risk exceeds 20% [52]. Our subjects had a median range of 1.0 - 7.5%. Limitations in the use of the Framingham tool for HIV-infected patients include: underestimation of cardiovascular events because key factors such as hypertriglyceridemia, are not used in the Framingham calculations. Also, there are direct effects of HIV and HAART on CVD risk that are not captured in the calculations [52]. Based on these, Law et al suggest that lower 10-year CVD risk calculations should be used to guide CVD interventions among HIV-treated patients than those used for the general population [53]. If this is adopted, maybe our subjects risk scores would fall within adverse ranges.

The cross-sectional design of this study was a limitation because a prospective study of HIV patients followed up after commencement of HAART would have enabled us to monitor their distinct metabolic changes. Also the Framingham risk calculator could not compute variables beyond certain limits.

## Conclusion

Considering the high prevalence of CVD risk factors in both the HIV naïve and HIV treated subjects observed in this study, it would be difficult to identify which patients are at risk for CVD simply from their HIV status or their antiretroviral therapy alone. It is suggested that regular biochemical examination of the cardiovascular system in all HIV-infected patients be performed to prevent atherosclerotic vascular complications and to reduce the risk for future cardiovascular events.

### What is known about this topic

- CVD is a leading cause of morbidity and mortality globally;
- Increasing prevalence of CVD risk factors predisposes the population to CVD;
- HAART use predisposes HIV treated patients to develop CVD risk factors.

### What this study adds

- The HIV virus itself predisposes untreated positive patients to develop CVD risk factors as well;
- Risk assessment of both naïve and treated HIV positive patients, which showed they are both at risk for future CVD.
